# The Genomic Impact of Selection for Virulence against Resistance in the Potato Cyst Nematode, *Globodera pallida*

**DOI:** 10.3390/genes11121429

**Published:** 2020-11-28

**Authors:** Kyriakos Varypatakis, Pierre-Yves Véronneau, Peter Thorpe, Peter J. A. Cock, Joanne Tze-Yin Lim, Miles R. Armstrong, Sławomir Janakowski, Mirosław Sobczak, Ingo Hein, Benjamin Mimee, John T. Jones, Vivian C. Blok

**Affiliations:** 1 Cellular and Molecular Sciences Group, James Hutton Institute, Dundee DD2 5DA, UK; ak.varypatakis@gmail.com (K.V.); miles.armstrong@hutton.ac.uk (M.R.A.); ingo.hein@hutton.ac.uk (I.H.); john.jones@hutton.ac.uk (J.T.J.); 2 School of Biology, University of St Andrews, North Haugh, St Andrews KY16 9TZ, UK; 3 Agriculture and Agri-Food Canada, 430 boul. Gouin, St-Jean-sur-Richelieu, QC J3B 3E6, Canada; pierre-yves.veronneau@canada.ca (P.-Y.V.); benjamin.mimee@canada.ca (B.M.); 4 School of Medicine, University of St Andrews, North Haugh, St Andrews KY16 9TZ, UK; pjt6@st-andrews.ac.uk; 5 Information and Computational Sciences Group, James Hutton Institute, Dundee DD2 5DA, UK; peter.cock@hutton.ac.uk (P.J.A.C.); tyinlim@gmail.com (J.T.-Y.L.); 6 School of Life Sciences, Division of Plant Sciences at the JHI, University of Dundee, Dundee DD2 5DA, UK; 7 Department of Botany, Institute of Biology, Warsaw University of Life Sciences (SGGW), Nowoursynowska 159, 02-766 Warsaw, Poland; slawomir_janakowski@sggw.pl (S.J.); miroslaw_sobczak@sggw.edu.pl (M.S.)

**Keywords:** potato cyst nematodes, *Globodera pallida*, next generation sequencing, target enrichment sequencing, whole genome sequencing, selection, virulence, effectors

## Abstract

Although the use of natural resistance is the most effective management approach against the potato cyst nematode (PCN) *Globodera pallida*, the existence of pathotypes with different virulence characteristics constitutes a constraint towards this goal. Two resistance sources, *GpaV* (from *Solanum vernei*) and *H3* from *S. tuberosum* ssp. *andigena* CPC2802 (from the Commonwealth Potato Collection) are widely used in potato breeding programmes in European potato industry. However, the use of resistant cultivars may drive strong selection towards virulence, which allows the increase in frequency of virulent alleles in the population and therefore, the emergence of highly virulent nematode lineages. This study aimed to identify *Avirulence* (*Avr*) genes in *G. pallida* populations selected for virulence on the above resistance sources, and the genomic impact of selection processes on the nematode. The selection drive in the populations was found to be specific to their genetic background. At the genomic level, 11 genes were found that represent candidate *Avr* genes. Most of the variant calls determining selection were associated with *H3*-selected populations, while many of them seem to be organised in genomic islands facilitating selection evolution. These phenotypic and genomic findings combined with histological studies performed revealed potential mechanisms underlying selection in *G. pallida*.

## 1. Introduction

Pests and pathogens of plants can engage in complex relationships with their hosts to establish conditions to enable survival and reproduction, whereas plants invest energy in their defence strategies to counteract them. During these interactions, pathogen-derived effectors promote virulence by suppressing or evading activated host defence responses. In some responses, specialised plant resistance (R) proteins that recognise effectors (or their biochemical activity) activate robust defence responses, including the hypersensitive reaction (HR) [1,2]. An effector recognised as part of a resistance response is termed an avirulence (avr) factor, and their interaction with the cognate *R* gene product is usually highly specific [2,3,4]. These interactions have been summarised as part of the zig zag model [2].

The potato cyst nematodes (PCN), *Globodera rostochiensis* (Woll.) and *G. pallida* (Stone), are economically important pests of potato. It has been estimated that 9% of global potato production is lost annually to these two species [5]. Today, both species are widespread, with Europe likely to have acted as a secondary source of their distribution for many areas of the world as a result of trade of infested seed potato [6]. Natural resistance is the most effective and environmentally benign way of controlling PCN [7,8]. Potato cultivars resistant to *G. rostochiensis* have been bred by the introgression of the major gene, *H1*, from *Solanum tuberosum* ssp. *andigena* CPC1673 (a potato clone from the Commonwealth Potato Collection). Several potential resistance sources to *G. pallida* have been identified in wild potato species, including *S. vernei*, *S. tuberosum* ssp. *andigena* [9,10], *S. multidissectum* [11], *S. spegazzinii* [12], *S. tarijense* [13], *S. sparsipilum* [14]. The degree of resistance they confer can vary and may diminish during the successive crosses that are performed to produce commercially attractive cultivars.

Both PCN species originate from South America and were likely brought into Europe during the 19th century [6,15]. Based on microsatellite and mitochondrial *cytochrome b* sequence analyses [16], several distinct introductions of *G. pallida* from different regions in Southern Peru into Europe (including the UK) seem to have occurred; by contrast, the *G. rostochiensis* present in the UK appears to have been derived from a more restricted introduction. The complex nature of the UK *G. pallida* populations [17] makes their control using host resistance potentially challenging as no single major *R* gene is likely to be effective and remain durable against the diversity of *G. pallida* present in Europe.

PCN are biotrophic pathogens that induce complex feeding structures in their hosts. Second stage juveniles (J2) invade roots and after migrating through cells towards the vascular tissues induce the formation of a large, metabolically active feeding structure, the syncytium. The syncytium has a reduced central vacuole, enriched cytoplasm and enlarged nuclei. It is formed by controlled breakdown of the walls between the initial syncytial cell targeted by the J2 and the surrounding cells followed by protoplast fusion [18]. This process is repeated with further layers of surrounding cells and several hundred cells may eventually be incorporated into the syncytium. Resistance against PCN is often mediated by a necrotic response surrounding the syncytium that prevents it from reaching the vascular tissues and thus limiting the nutrients available to the developing nematodes [19,20].

The interactions between *G. pallida* and their host can lead to adaptations that allow the nematodes to evade particular resistance *loci* [10,21]. Changes have been observed in the genomic background of populations [22,23] and, depending on the exerted selection pressure, a directional change in a specific *locus* (e.g., single nucleotide polymorphisms - SNPs, indels, deletions etc.) may occur in favour of virulence [24]. Successive multiplication of populations on resistant genotypes can give rise to selection for virulent (*vir*) alleles that are already present at low levels in the initial genetic pool; this can result in specific resistances being overcome [10,25,26,27].

Because of the key roles that they play in the interactions between nematodes and their host plants, significant effort has been put into identifying and characterising the effectors of plant-parasitic nematodes, including cyst nematodes. Analysis of expressed sequenced tags (EST) from cDNA libraries was, until recently, the primary approach used to study and identify plant-parasitic nematode effectors [28,29]. However, the publication of the genome sequences of many plant-parasitic nematodes, including *G. pallida* [30] and *G. rostochiensis* [31] has allowed whole effector complements from these nematodes to be characterised [32].

The aim of the present study was to investigate the impact of selection on host resistances from *S. vernei* or *S. tuberosum* ssp. *andigena* CPC2802 (alternatively *H3*) [9,10,33] on the genomic background of *G. pallida* populations and to identify candidate *Avr* genes that are the targets of this selective pressure. Two genome-based approaches were combined; a targeted approach for specifically capturing and sequencing effector-encoding genes (i.e., pathogen enrichment sequencing—PenSeq) [34], and a whole-genome scanning (re-sequencing—ReSeq) of host-adapted nematode populations. In addition, histological analysis of the nature of the resistance response induced in plants carrying the *H3 R* gene when infected with *G. pallida* was performed.

## 2. Materials and Methods

### 2.1. G. pallida Populations

The *G. pallida* populations from The James Hutton Institute PCN collection used were Lindley, Newton and two sub-populations generated by selection on partially resistant germplasm (n-11305 and n-11415). Both sub-populations were obtained following 12 generations of multiplication on potato genotypes with resistance derived from *S. vernei* (Sv_11305—“Morag”) or *S. tuberosum* ssp. *andigena* CPC2802 (Sa_11415), respectively, as described in Phillips and Blok [10]. The Newton population was maintained on the susceptible cultivar “Désirée”.

### 2.2. Histological Analysis of the H3 Resistant Response

Susceptible potato (*S. tuberosum*) cv. “Pentland Ivory” and Sa_12601 (a breeding line derived from *S. tuberosum* ssp. *andigena* CPC2802 (*H3*) [35] were used for the histological analysis. Three weeks following planting of a single-sprouted tuber piece (≈ 1.5 cm × 1.5 cm) in 10 cm pots containing sterile sand:loam 1:1, 6 pots for each potato genotype, each pot was inoculated with about 400 freshly hatched 2nd-stage juveniles (J2s) of *G. pallida* population Lindley. Two, 4 and 7 days post inoculation (dpi), 2 pots were selected and the roots were removed from the soil, washed briefly under tap water and the root system was immersed in a fixative (2% (*w/v*) paraformaldehyde, 2% (*v/v*) glutaraldehyde, 0.05 M cacodylic buffer, pH 7.2) at room temperature. The samples consisting of root segments with attached juveniles were dissected under a stereo microscope and transferred into fresh fixative. They were fixed for 2 h and thereafter rinsed 4 times with 0.05 M cacodylic buffer. They were post-fixed in 2% (*w/v*) osmium tetroxide and successively dehydrated with a series of aqueous ethanol solutions, substituted with propylene oxide, infiltrated with Araldite resin (Sigma-Aldrich, St. Louis, MO, USA), embedded in flat embedding moulds and polymerised [36]. Semi-thin sections (3 μm thick) were stained with hot 1% (*w/v*) aqueous solution of Crystal Violet (Sigma-Aldrich) and examined under an Olympus AX70 “Provis” (Olympus, Tokyo, Japan) light microscope equipped with an Olympus DP50 digital camera. Ultra-thin sections (80–100 nm thick) were collected on formvar-coated (Sigma-Aldrich) grids and stained in a saturated methanolic solution of uranyl acetate and aqueous solution of lead citrate. They were examined under an FEI M268D “Morgagni” (FEI Corp., Hillsboro, CA, USA) transmission electron microscope operating at 80 kV and equipped with an SIS “MegaView III” (Soft Imaging Systems, Münster, Germany) digital camera. Gained images were equalised for similar contrast and brightness, cropped, and resized using Adobe Photoshop (Adobe Inc., San Jose, CA, USA) software. Between 5 and 13 samples were examined for each timepoint and genotype.

### 2.3. Comparison of G. pallida Reproduction on 3 Potato Genotypes and Collection of Females

The same nematode populations described above were used to produce adult-stage female nematodes for both PenSeq and ReSeq analyses. Five potato genotypes were used: cv. Désirée (susceptible), Sv_8906, Sv_11305, Sa_11415 and Sa_12674 (Sv_8906 and Sv_11305 have resistance derived from *S. vernei*, while Sa_11415 and Sa_12674 have resistance derived from *S. tuberosum* ssp. *andigena* CPC2802 [10]. Multiplication was performed in a greenhouse (20 °C/16 °C day/night, 8 h continuous dark conditions) in 12 cm-deep root trainer cells (Haxnicks, Bristol, UK) filled with insecticide-free compost. The soil in each cell was inoculated with 20 cysts from population n-11305 or n-11415 and then a single-sprouted tuber section (≈1.5 cm × 1.5 cm) from one of the five examined potato genotypes was planted. Each “population × potato genotype” combination consisted of four biological replicates organised in a completely randomised design and the test was performed twice. Eight weeks post-inoculation, the females visible on the root surface were counted, collected with fine tip tweezers, and placed into an Eppendorf tube, which was immediately frozen in liquid nitrogen.

### 2.4. DNA Extraction and Quantification

The DNA used in both genomic approaches was extracted from 40 females of each of n-11305 collected from Sv_8906 and Sv_11305, and n-11415 collected from Sa_11415 and Sa_12674. In the PenSeq analysis, DNA from unselected Newton population grown on cv. Désirée was also included as a baseline comparison.

Mechanical crushing was achieved with sterile micro-pestles in 600 μL cell lysis buffer (Qiagen, Hilden, Germany) and 5 μL proteinase K (20 mg mL^−1^) (Qiagen). To allow maximum tissue lysis, the samples were incubated for 18 h at 56 °C. Lysates were treated with 4 μL RNAse A (100 mg mL^−1^) (Qiagen) followed by 10-min incubation at room temperature. To precipitate proteins, 200 μL precipitation buffer (Qiagen) was added to each sample followed by centrifugation at 16,500× *g* at 4 °C. The supernatant was transferred to a new tube where 600 μL isopropanol (Sigma-Aldrich) and 0.25 μL glycogen (20 mg mL^−1^) were subsequently added. DNA precipitation was done overnight at −20 °C. The precipitated DNA was isolated after a 45-min centrifugation of the overnight-incubated samples at 19,000× g at 4 °C and washed by re-suspending the pellet in 600 μL 70% ethanol. Ethanol was removed after a 30 min-centrifugation. The remaining pellet was air-dried at room temperature and re-suspended in 21 μL elution buffer AE (10 mM Tris-Cl, 0.5 mM EDTA) (Qiagen).

Quantification was done using a Qubit^TM^ 3.0 fluorometer (Thermo Fisher Scientific, Waltham, MA, USA) following the manufacturer’s instructions.

### 2.5. Statistical Analysis

Statistical analysis was conducted in R [37]. Data were modelled and models were subsequently checked visually for normality between the two different experiments. Non-significant values were dropped from the model and transformations applied if necessary.

### 2.6. Targeted Sequencing, Library Preparation and Enrichment (PenSeq)

#### Bait Design

Biotinylated RNA-based 120 nt-long oligos were designed to fully cover the target genes representing all potential effectors in *G. pallida* using a 2× tilling density. In total, 24,744 baits were designed and manufactured by MYcroarray MYbaits (Arbor Biosciences, Ann Arbor, MI, USA) to target 700 target genes (Supplementary Table S1), which were composed of previously characterised effector genes from *G. pallida* [32], effector genes that are downstream of the gland promoter DOG box motif [31] and *G. pallida* orthologues of characterised effectors from other, closely related plant-parasitic nematode species.

### 2.7. PenSeq Library Preparation and Sequencing

DNA samples from the 5 nematode and potato genotype combinations, in duplicate, were used. Five nanograms of DNA from each sample was sheared in a M220 ultrasonicator (Covaris, Woburn, MA, USA) to produce ~500 bp fragments using the following conditions: Peak incident power 50 W, 200 cycles per burst, 20% duty factor for 60 s.

For the library preparation, the NEBNext^®^ Ultra^TM^ DNA library prep kit for Illumina^®^ (New England BioLabs, Ipswich, MA, USA) was used. Each library was amplified with a unique and specific index (barcode) provided in the NEBNext^®^ Multiplex Oligos for Illumina^®^ kit (Index Primers Set 1) (New England BioLabs). After library preparation, the samples were checked on a 2100 Bioanalyzer (Agilent Technologies, Santa Clara, CA, USA) to confirm the fragment sizes. Equimolar amounts of DNA from the 10 individually barcoded samples were pooled to obtain 500 ng of total pooled DNA. Enrichment was performed according to the guidelines provided in the MYcroarray MYbaits kit (user manual v3) (Arbor Biosciences). Enrichment hybridisation was performed at 65 °C for 22 h. The post-capture amplification was achieved using the Herculase II polymerase (5 U μL^−1^) (Agilent Technologies). Purification of the samples was performed after shearing, indexing and the final post-capture amplification using AMPure^®^ XP beads (Beckman Coulter, Brea, CA, USA) and following the manufacturer’s instructions. Quantification of DNA was done on a Qubit^TM^ 3.0 fluorometer instrument (Thermo Fisher Scientific).

Sequencing of the targeted pooled, enriched library was done on the Illumina^®^ MiSeq^TM^ platform at The James Hutton Institute using the v2 reagent kit and 2 × 250 bp conditions. All the raw sequencing data have been archived in the European Nucleotide Archive (ENA) (http://www.ebi.ac.uk/ena/data/view/PRJEB41175).

### 2.8. Variant Calling of the PenSeq Samples

Quality control was done on the Illumina^®^ MiSeq-generated raw reads using the tool FastQC v0.11.7 [38]. All the reads were trimmed with Trimmomatic v0.38 [39] to a minimum length of 36 bp using Phred quality score at 33. Alignment of the trimmed reads was performed with bowtie2 v2.3.4.2 [40] keeping the minimum acceptable alignment score set to “L, −0.12, −0.12”. A new *G. pallida* genome assembly generated from the Newton population was used as the reference genome for mapping [41] excluding the secondary alignments. Variant calling was done using FreeBayes v1.2.0 [42] with the minimum mapping quality set to 20, minimum alternate count to 5 and minimum alternate fraction to 0.2. The main filtering for minimum read coverage of 10 and differences between the biological groups (i.e., populations selected on the same resistance) was done using PyVCF (https://github.com/jamescasbon/PyVCF), and generated variant were annotated based on their putative effect with SnpEff v4.3 [43]. Only variant calls with high and moderate impact were kept. Blast2GO [44] was used to BLAST (Basic Local Alignment Search Tool) search all the amino acid sequences with a contained variant for gene prediction.

### 2.9. Real-Time PCR (qPCR) Analysis of Enriched Samples

The enriched captured DNA was tested with qPCR, using primers that amplify three known effector genes (*SPRY-414-2*, *SPRY-1719-1*, *G16H02*; [32]). The non-effector *GAPDH* (*Glyceraldehyde 3-phosphate dehydrogenase*) gene was used as an endogenous control. Levels of the DNA encoding these target sequences was compared with those in the pre-enrichment libraries. All the primers used are shown in Supplementary Table S2.

Two DNA templates were used; the pre-enrichment and post-enrichment pooled equimolar libraries. Three replicates were prepared for each sample using standard procedures and were run on a StepOne real-time PCR instrument (Thermo Fisher Scientific) (quantitation comparative CTΔΔCT). Data were analysed using StepOne Software (version 2.3) (Thermo Fisher Scientific).

### 2.10. Whole-Genome Scanning (ReSeq)

#### Library Preparation for ReSeq and Sequencing

DNA samples from the four nematode population and potato genotype combinations were used. A total of 150 ng of DNA from each sample was used for library preparation with the NEBNext^®^ Ultra™ II DNA Library Prep Kit (New England BioLabs) at the McGill University and Genome Quebec Innovation Centre (McGill University, Montreal, QC, Canada). The individual population libraries were indexed with IDT (Coralville, IA, USA) dual-index oligos and checked for fragment size and quality on a 2100 Bioanalyzer (Agilent Technologies). Then 200 pM of each of the four libraries were pooled and sequenced on a single lane of Illumina^®^ HiSeq4000 using 2 × 100 bp reads. Likewise PenSeq, the HiSeq-generated raw sequencing data have also been archived in the ENA database (http://www.ebi.ac.uk/ena/data/view/PRJEB41175).

### 2.11. Variant Calling of the ReSeq Samples

Curation and trimming of the Illumina^®^ HiSeq-generated reads was performed as described above. Alignment was performed with BWA v0.7.12 [45] against the new *G. pallida* genome assembly [41] using the default settings. Variant calling was performed with FreeBayes v1.2.0 [42] with minimum mapping quality set at 30, minimum base quality at 20, minimum alternate count at 2, minimum alternate fraction at 0.05. Only the variant calls with minimum coverage of 10 were kept.

Variants were screened to keep only those that behave in a similar way in populations selected on the same resistance source (up to 30% difference in their allele frequency (AF)) and differently (at least 70% difference in AF) between populations selected on the different resistances. SnpEff v4.3 [43] was used to annotate the identified variants based on their putative effect; only the calls with high and moderate impact were kept for the analyses. Blast2GO [44] was used to BLAST search all the amino acid sequences with a contained variant for functional annotation.

Distribution histograms of the identified variants on the genome were generated using a java-built software (written by Etienne Lord, Agriculture and Agri-Food Canada, St-Jean-sur-Richelieu, QC Canada) (Supplementary Script) and built with Processing (https://processing.org/), based on the *G. pallida* genome FASTA file, as well as the VCF file with the identified variants.

## 3. Results

### 3.1. Histological Analysis of the Resistant Response to H3

**Figure 1:**
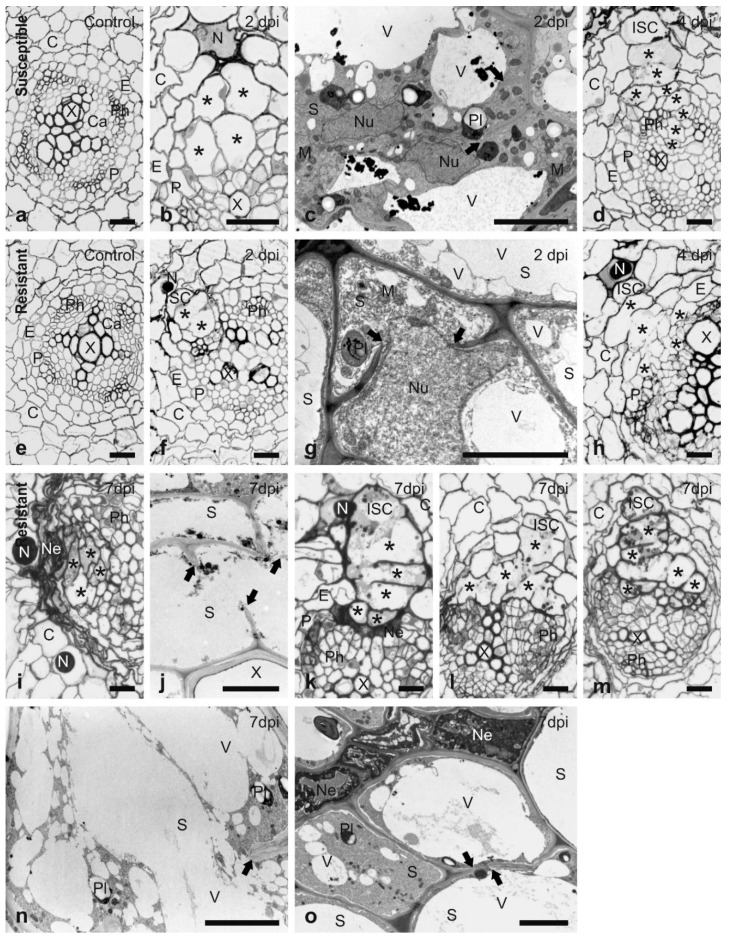
Development of syncytia induced by *G. pallida* in the roots of susceptible and resistant potato genotypes. Bright field light microscopy (**a**,**b**,**d**–**f**,**h**,**i**,**k**–**m**) and transmission electron microscopy (**c**,**g**,**j**,**n**,**o**) micrographs taken from tangential sections of susceptible *S. tuberosum* cv. Pentland Ivory (**a**–**d**) and Sa_12601 (resistance from the Commonwealth Potato Collection (CPC) *S. tuberosum* ssp. *andigena* CPC2802 (*H3*)) (**e**–**o**) roots. (**a**) Uninfected root of susceptible genotype; **b**,**c**, root of susceptible genotype with syncytia at 2 dpi; (**d**), root of susceptible genotype with syncytium at 4 dpi; **e**, uninfected root of Sa_12601; (**f**,**g**), root of Sa_12601 with syncytia at 2 dpi; **h**, root of Sa_12601 with syncytium at 4 dpi; (**i**–**o**), root of Sa_12601 with syncytia at 7 dpi. *Abbreviations:* C, cortex; Ca, pro-/cambium; E, endodermis; ISC, initial syncytial cell; M, mitochondrion; N, nematode; Ne, necrosis; Nu, nucleus; P, pericycle; Ph, phloem; Pl, plastid; S, syncytium; X, xylem; V, vacuole. Asterisks (*) indicate selected syncytial elements; arrows point to cell wall stubs. Scale bars: 20 µm (**a**,**b**,**d**–**f**,**h**,**i**,**k**–**m**), 5 µm (**c**,**g**,**j**,**n**,**o**).

In order to confirm that *H3*-mediated resistance is based on a typical *R* gene response rather than a general toxicity to invading nematodes, the histology and ultrastructure of the interaction were compared between *G. pallida* and the susceptible *S. tuberosum* cv. Pentland Ivory with that of Sa_12601, which contains the resistance derived from *S. tuberosum* ssp. *andigena* CPC2802 (*H3*). Sections taken at the upper region of root-hair zones of susceptible and resistant genotypes showed the same anatomical organisation of uninfected roots (Figure 1a,e). The roots consisted of a single cell layer of epidermis, three cell layers of cortical parenchyma and a single cell layer of endodermis. The vascular cylinder was tetrarchic or triarchic with the outermost single layer of the pericycle. The primary growth of the root at this region was completed and cambial strips began to form between primary xylem and phloem elements.

In roots of both genotypes, the nematode juveniles were usually found with their heads located among outer cortex parenchyma cells (Figure 1b,f) at 2 dpi. These cells were usually selected as initial syncytial cells (ISC). Only rarely did they migrate deeper into the root and select ISCs from among inner cortical parenchyma, endodermal, pericyclic or pro-/cambial cells. When the ISC was selected among cortex cells, the syncytium expanded from the ISC toward the vascular cylinder by incorporation of slightly enlarged cortical, endodermal and pericyclic cells creating a so-called “cortex bridge” [46]. The development of syncytia in Sa_12601 was faster than in the susceptible cv. Pentland Ivory (Figure 1f, b), but no incorporation of pro-/cambial cells into syncytia was observed at this stage. At the ultrastructural level, the cytoplasm in syncytia induced in susceptible plants was strongly proliferating, the nuclei became enlarged and amoeboid. Furthermore, the numbers of mitochondria, plastids with starch grains and endoplasmic reticulum structures increased, whereas the volume of central vacuoles decreased and they were often divided into smaller vacuoles (Figure 1c). In contrast, proliferation of the cytoplasm in syncytia induced in resistant plants was weak; it was located only paramurally and had a granular appearance when observed by transmission electron microscopy (Figure 1g). The nuclei were enlarged and turned amoeboid, but the nucleoplasm was granular and relatively more electron translucent in comparison to syncytia induced in susceptible plants. Similarly, the stroma of plastids and the matrix of mitochondria were also more electron translucent than in syncytia induced in resistant plants. Except for the central vacuoles, numerous small vacuoles were formed in the syncytial cytoplasm.

Syncytia induced in susceptible plants incorporated many enlarged pro-/cambial cells and expanded into the centre of the vascular cylinder between the xylem and phloem bundles at 4 dpi (Figure 1d). In contrast, syncytia induced in resistant plants did not incorporate pro-/cambial cells and their expansion was stopped at the border of the pericycle (Figure 1h). In the region close to the nematode head, the syncytia were composed of several slightly enlarged “cortex bridge” elements and a handful of relatively strongly enlarged pericyclic cells (Figure 1h). However, the distal regions of syncytia expanding along the vascular cylinder were composed of only strongly enlarged pericyclic cells, thus, the syncytia had a collar-like shape in these regions. Most of the syncytia examined contained protoplasts that were ultrastructurally similar to those observed in 2 dpi syncytia, whereas the others contained only degraded and strongly osmiophilic remnants of syncytial cytoplasm (as depicted for 7 dpi syncytia shown in Figure 1j). Necrosis occurred only in a few cells next to the juveniles that were probably mechanically destroyed during nematode migration and no degraded cell was found around the syncytium at this stage.

At least four different types of syncytium organisation were found in samples collected from roots of resistant plants at 7 dpi. Most frequently the syncytia were degraded and compressed, and concomitantly neighbouring cortex parenchyma and endodermal cells were also degraded and collapsed forming extensive necrosis (Figure 1i). Syncytial protoplasts were usually degraded and only osmiophilic remnants were present (Figure 1j). However, in a few cases, the syncytia still contained granular, electron translucent and strongly vacuolated protoplasts, which were similar to those observed in 2 and 4 dpi syncytia (Figure 1n). In the second type, the syncytia were composed of elements derived from cortical and endodermal cells only (Figure 1k) and they were separated from the vascular conductive tissues by a layer of degraded cells. These syncytia contained a thin layer of paramurally located electron-dense cytoplasm with a very limited number of plastids, mitochondria and endoplasmic reticulum structures (Figure 1o). The third type encompasses syncytia composed of cortical, endodermal and pericyclic cells and which did not expand into the centre of the vascular cylinder (Figure 1l). They were not surrounded by degraded cells, but their protoplast was electron-translucent and strongly vacuolated (similarly as depicted in Figure 1n). Finally, a single syncytium (among 13 examined) was found that apparently incorporated pro-/cambial cells from the vascular cylinder tissues (Figure 1m) and expanded inside the vascular cylinder. It contained a thin layer of electron-dense cytoplasm (similar to that seen in Figure 1o) and no cell degradation occurred around this syncytium.

### 3.2. The Reproductive Ability of Selected Nematode Populations Is Dependent on the Genetic Background of the Potato Genotype

The three *G. pallida* populations were challenged on Sv_8906, Sv_11305, Sa_11415 and Sa_12674 to assess their reproductive ability in the presence of the resistance sources derived from *S. vernei* and *S. tuberosum* ssp. *andigena* CPC2802 (*H3*) and compared to that on the susceptible “Désirée” (Table 1). All selected populations showed high reproductive ability on the susceptible “Désirée” (from approximately 84 to 150 females per plant). The *S. vernei*-selected population, n-11305, showed approximately three times higher reproductive ability (*p* = 0.04) (compared to the unselected Newton) when tested on Sv_8906 containing the same resistance as it had been selected on originally, whereas the *H3*-selected population n-11415 did not show increased virulence on Sv_8906 when compared to Newton (*p* = 0.39). Similarly, n-11305, which was also selected on *S. vernei,* displayed significantly increased multiplication on Sv_11305 (i.e., 4.6 times; *p* < 0.001) when compared to the Newton population, and multiplication of the *H3*-selected n-11415 was not significantly increased (*p* > 0.1).

With the potato clones that have *H3*-derived resistance, the population n-11415 showed an approximately 4-fold greater multiplication on Sa_11415 (*p* < 0.001), whereas n-11305 showed no statistically significant increased multiplication (*p* > 0.1) when compared to Newton. When they were challenged on the Sa_12674 potato clone, the population sharing the same selection background (i.e., n-11415) displayed 2.6 times (*p* > 0.1) higher reproductive ability compared to the Newton population, whereas the *S. vernei*-selected n-11305 had a reduced reproductive ability. However, in both cases, these differences were not statistically significant (*p* = 0.9).

Selection, therefore, resulted in a specific increase in virulence against the resistance source used rather than a general increase in aggressiveness or infectivity.

### 3.3. Post-Enriched Captured Libraries (PenSeq) Are Highly Enriched for Known Effectors

A real-time PCR (qPCR) was performed on the pre- and post-enrichment libraries using primer pairs (Supplementary Table S2) that amplify three known effectors (SPRY-414-2, SPRY-1719-1 and G16H02) in *G. pallida*. All three effector genes showed an approximate 100-fold to 1000-fold increase in amplification in the post-enrichment compared to the pre-enriched library (Supplementary Figure S1). Each of the effector gene was present at very low levels in the pre-enrichment library. This analysis confirmed that the post-enrichment library was highly enriched for these sequences.

### 3.4. Genomic Analyses Allow Identification of High Confidence Variant Genes

The enrichment sequencing analysis generated 23,590,596 paired-end Illumina^®^ MiSeq^TM^ reads (97.5% of which passed the filtering criteria), of which 9,272,706 assembled paired-end reads were retained after adapter and quality trimming. In total, 481 variant calls were identified, with the majority being SNP (Supplementary Table S3). Approximately 1/3 of the variants caused synonymous changes and therefore, no alteration in the encoded amino acid sequence. By analysing the putative impact on the gene products of non-synonymous changes, 285 variant calls were found on annotated genes. From those, 139 had high or moderate functional impact on the gene product and were located in 85 variant genes (Supplementary Table S4). From the total number of the retained calls (high and moderate impact), one caused a premature stop codon (“stop gained”), three changed the translational reading frame (“frameshift variant”) and the remainder resulted in a different amino acid (“missense variant”) (Supplementary Table S4).

The four *G. pallida* populations multiplied on Sv_8906, Sv_11305, Sa_11415 or Sa_12674 (see also Table 1) were whole genome scanned on Illumina^®^ HiSeq^TM^. A total of 365,204,135 paired-end reads were obtained from which 3,466,822 variants were detected initially, and 1,178,090 were retained after the first filtering steps (i.e., min DP of 10). From these, 871,459 were SNPs and 181,194 were indels (i.e., inserts or deletions). Approximately 20% were identified in exons or introns of annotated genes and thus, were kept for analysis. After the filtering steps based on the AF value differences, 21,162 variants in total were retained (Supplementary Table S5), of which 19,592 caused a predicted modification of function, i.e., non-synonymous variants. According to the SnpEff annotation [43], 1380 variant calls had high- or moderate-impact on their putative impact on a total of 668 predicted gene products (full list in Supplementary Table S6), which differ between the populations selected on *S. vernei* or *H3*. Most of the calls with high impact caused a change in the translational reading frame (in total 53 calls) (“frameshift variant”), while 16 had an impact on the start (“start lost”) or stop (“stop gained”, “stop lost”) codon. One variant call was predicted to be in the splice site (“splice acceptor variant”) on the SPRY family gene *GPALN_015102*.

### 3.5. Proteins Containing a SPRY Domain Were the Most Abundant Identified as Variants in the Selected Populations

In line with the bait design that included a high number of SPRY, (SP1a and Ryanodine receptor) family members (PF00622), these genes were highly abundant (45 genes out of 139) after PenSeq analysis and variant calling (Supplementary Table S4). SPRY domain family consists of several hundred different sequences in *G. pallida* and a subset of these includes a signal peptide (i.e., SPRYSECs) and are deployed as effectors by the nematode [47]. One SPRYSEC, *RBP-1*, has been identified as the *Avr* gene recognised by the *Gpa2* resistance gene [4].

Polymorphisms were also identified in several other characterised effectors including three cellulases (PF00150) (GPALN_010075, GPALN_014324, GPALN_016072), glutathione synthase (GPALN_002202; PF03199), an orthologue of *Heterodera glycines* GLAND3 (GPALN_015425, previously known as G12H04), a D406 (GPALN_009695) (similar to *H. glycines* GLAND5) and GPALN_006112 with unknown function (Supplementary Table S4).

### 3.6. Most of the Identified Variants in Both Genomic Approaches Were Associated with H3-Selected Populations

**Figure 2:**
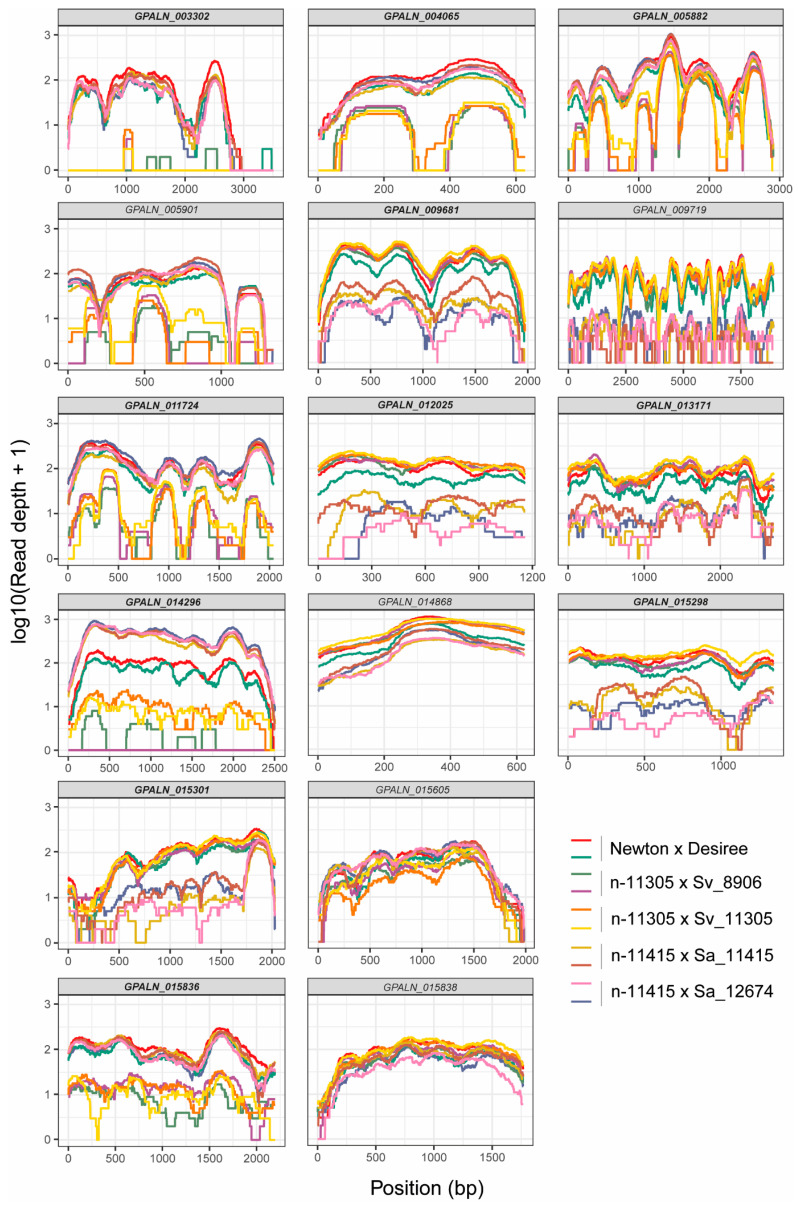
PenSeq analysis of candidate effector genes on the tested *G. pallida* populations. Each box represents an entire reciprocal best blast hit (RBBH) coding sequence of a gene (including their exons) (*x*-axis). The *y*-axis indicates the coverage of the targeted gene on a log-scale. Each line shows the coverage of a certain nematode population. Genes with full coding sequence on nematode populations selected on a certain resistance source only are represented in bold.

Analysis of reads coverage of targeted PenSeq candidate genes showed that selection can be very specific to the selection background of a nematode population, and that some effector genes can be lost during selection evolution (Figure 2) (Supplementary Figure S2).

In the variant genes identified during PenSeq analysis, 81 were associated with the *H3*-selected *G. pallida* populations and 58 with *S. vernei*-selected populations. As was mentioned above, in the re-sequenced libraries, 668 candidate variant genes were identified in total, of which 52% (344 genes) contained variants in populations developing on *H3* and 41% (275 genes) in populations developing on *S. vernei*. When both lists with variant genes identified were cross-referenced, 13 genes were identified in common, and these are presented in Table 2. Two of them (*GPALN_006664* and *GPALN_010968*) showed inconsistent results between the two genomic approaches suggesting they may be artefacts. From the final 11 candidate variant genes in common, 4 were associated with *S. vernei* resistance (*GPALN_004071*, *GPALN_004129*, *GPALN_013052* and *GPALN_013436*) and 7 with *H3* resistance (*GPALN_007439*, *GPALN_009681*, *GPALN_009697*, *GPALN_012007*, *GPALN_012018*, *GPALN_015286* and *GPALN_015295*). Finally, 49 variant genes in ReSeq had variant calls on the populations associated with both resistances (Supplementary Table S6).

### 3.7. Variant Genes with Complete Drop of the Reference Allele Represent Potential Avr Genes

Forty-eight variant calls in the ReSeq were homozygous for the alternate allele. From these, 25 variant calls (located in 16 genes) and 23 (in 14 genes) were on *S. vernei*- and *H3*-selected *G. pallida* populations, respectively (Supplementary Table S6). Except for two variant calls, all were annotated as missense variants. The remaining two caused a change in the reading frames (“frameshift variant”). About half of the calls were in annotated genes with unknown function. Additionally, 3 genes also contained variants associated with populations of the other resistance source. Specifically, in each of the genes *GPALN_005885* and *GPALN_015793*, there was a SNP homozygote for the alternate allele in the *S. vernei*-selected populations and an additional mutation on the *H3*-selected populations (with AF values varied from 0.16 to 0.04). Similarly, in the gene *GPALN_012150*, in addition to a complex mutation with a complete drop of the reference allele in the populations selected on *H3*, a SNP (with AF values between 0.39 and 0.18) was found on the populations selected on *S. vernei* (Supplementary Table S6).

### 3.8. Variants Are Not Located Randomly in the G. pallida Genome

**Figure 3:**
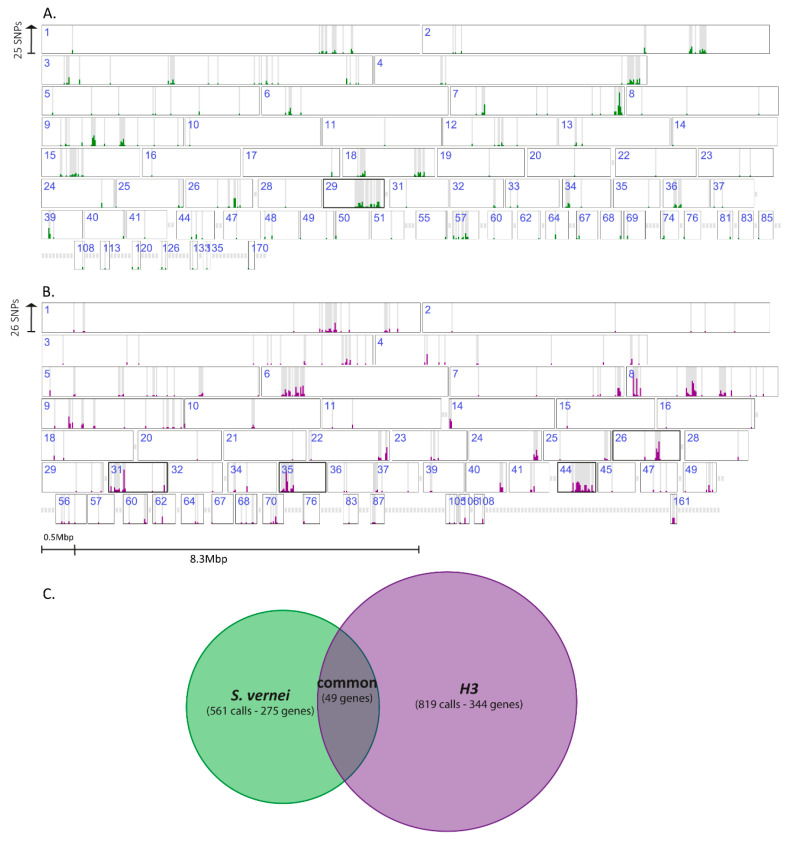
Distribution of variant calls (with high and moderate impact) identified in the ReSeq analysis in the (**A**) *S. vernei*- and (**B**) *H3*-selected *G. pallida* populations. The height of the bar (green—calls associated with *S. vernei*-selected, purple—calls associated with *H3*-selected populations) is relative to the maximum distribution of the identified single nucleotide polymorphisms (SNPs) in a specific region ((**A**) max distribution is 25 SNPs and (**B**) max distribution is 26 SNPs). Each box represents each scaffold of the *G. pallida* genome assembly [41]. Scaffolds with no identified filtered variant calls are not presented. Scaffolds with highlighted outline are potential genomic islands. (**C**) Venn diagram of the number of filtered calls and genes selected in the two biological groups.

Variants identified in ReSeq were located in 134 of the 173 scaffolds of the *G. pallida* genome. In some scaffolds, they were scattered along the whole length (e.g., in scaffolds 3 and 9), while in other scaffolds the variant calls were focused in very specific regions (e.g., in scaffolds 26 and 44) forming islands (Figure 3). A closer examination of some of these regions revealed that some scaffolds showed specific accumulation of variants associated with one of the resistances. For example, small regions of the scaffolds 29, 31, 35 and 44 showed high proportions of variants in a relatively small region. Remarkably, 9 out of 10 of these were associated with populations selected on one or the other of the resistance sources. For example, scaffold 29 was associated mainly with *S. vernei*-selected populations, whereas scaffolds 31, 35 and 44 were associated with *H3*-selected populations (Figure 3).

**Figure 4:**
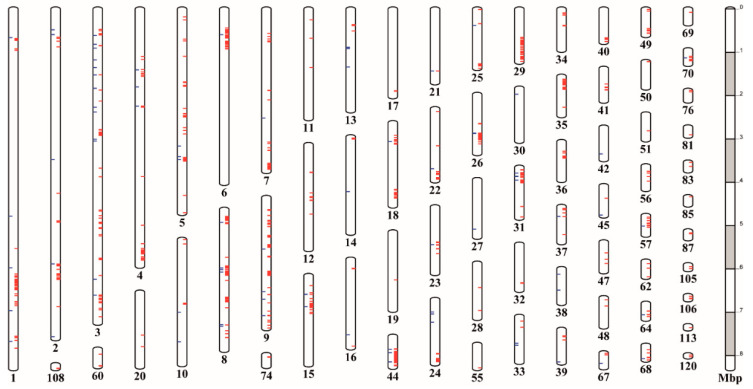
Representation of the physical position of the variant calls identified in PenSeq (blue lines) and ReSeq (red lines) along the *G. pallida* genome. Each box represents a scaffold of the *G. pallida* genome [41]. On the right, the scale shows the scaffolds size (in Mbp).

In the ReSeq analysis, many identified variant genes were present inside the genomic islands identified in the PenSeq analysis. In total, 66 genes were annotated in scaffolds 31, 35 and 44. Fifteen of them belonged to the SPRY family, two of them were orthologues of genes from other plant-parasitic nematode species that are known to be expressed in the dorsal gland cells (GPALN_010237, GPALN_012026), four contained a zinc finger domain (C2H2 or C3H4 types—PF00096, PF13920, respectively) (*GPALN_010234*, *GPALN_012013*, *GPALN_012039* and *GPALN_012044*) and one encoded a putative effector (*GPALN_012025*) with unknown function. Notably, all but one showed strong selection only with the populations developed on *H3* (i.e., n-11415 and n-12674). On the other hand, 28 out 31 variant genes found in scaffold 29 were mostly selected with the *S. vernei*-grown populations, n-8906 and n-11305. All the variants identified in both genomic analyses are presented in Figure 4.

Although few of the variant genes identified in the populations selected in both resistances (Supplementary Table S6) were located in some of the islands, there is evidence that these specific genomic regions can function as focal points for genetic selection during biological selection for virulence. Identified members of specific protein families contain variants from selection towards a specific resistance source. From the 178 variant calls found on SPRY domain genes, 147 (i.e., 62 SPRY domain genes out of 85) were associated with the populations developed on *H3*. Similarly, BTB/POZ domain proteins (PF00651) were mostly enriched with *H3*-selected populations as were two cellulases (β-1,4-endoglucanase, GHF5 glucanase) (PF00150) that were also identified during the PenSeq. On the other hand, most of the mutations on zinc finger domain proteins (PF00096) were associated with *S. vernei*-selected populations, whereas those in dorsal gland-expressed genes seem to be associated with both resistances (Supplementary Table S6).

## 4. Discussion

Since their introduction into Europe, *G. pallida* populations have been spread widely, and the proliferation of this species has been exacerbated by the paucity of potato cultivars with resistance to *G. pallida* [32,48]. Resistance currently available against *G. pallida* is quantitative, and virulence against these resistance sources can be selected for following repeated rounds of multiplication [10,21].

The nature of the response towards *G. pallida* in plants containing the *S. vernei* resistance source has been studied [49]. This response includes a strong necrotic reaction around the migrating and sedentary J2 as well as a degradation of the syncytium protoplast that leads to a failure of most nematodes to develop beyond the J3 stage at 10 dpi. However, no information on the nature of the response generated by the *H3 R* gene from *S. tuberosum* ssp. *andigena* CPC2802 against *G. pallida* is currently available. Microscopic examination of anatomy of *G. pallida*-infected roots indicates that, mechanisms leading to induction and development of syncytia formed in roots of resistant and susceptible solanaceous plants by *G. pallida* and *G. rostochiensis* have a lot in common [19,20,36,46,49,50,51,52]. Except for a single report concerning the resistance response of *S. canasense* plants, no delay in root invasion after nematode inoculation has been reported [52]. When juveniles enter tomato or potato roots, they preferentially select the ISC from among the outer cortex layers. From the ISC, syncytia expand towards the vascular cylinder by formation of so-called “cortex bridge” composed of cortex parenchyma and endodermal cells [46]. Reaching the vascular cylinder, pericyclic cells and procambial or cambial cells are also incorporated into the syncytium and it expands into the centre of the vascular cylinder. Thus, they secure a direct and extensive contact interface with conductive elements, where nutrients and water are transported. Next, the syncytium expands along the root axis via incorporation of the vascular cylinder cells only [19,20,46,49,50,51].

In *Globodera*-resistant genotypes, this developmental pattern is usually disturbed when the growing syncytium meets the vascular cylinder. Frequently, vascular cylinder cells in contact with the syncytium start to degrade and form a layer of necrotised cells preventing the syncytium from having direct contact with conductive elements [19,20,36]. This results in successive degradation of syncytial protoplasts and starvation, or obstruction of the development of associated juveniles, or the predominance of male nematodes. Occasionally, the degradation of syncytium is not followed by a formation of a layer of necrotic cells, as in the case of resistance from *S. vernei* [49].

Resistance provided by the *H3* gene from *S. tuberosum* ssp. *andigena* CPC2802 against *G. pallida* seems to be based on the same mechanism of defence as described for *S. vernei* at least until 4 dpi. Syncytia are formed, which are composed primarily of cortex cells, the quality of the cytoplasm and organelles is poor and many of them have completely necrotised protoplasts by 4 dpi. Cells with degraded protoplasts are present only around nematode migration paths and sedentary ones. However, extensive necroses of cortex cells was found at 7 dpi and large regions of the root cortex were completely collapsed in most of the samples examined. These syncytia may be associated with juveniles stopped in the J2 developmental stage. However, there are a few syncytia that are not affected by this extensive necroses at 7 dpi and still contain non-lethally degraded protoplasts, which might provide enough nutrients and function long enough to support development of J3, or even adult males.

The histological examination of roots from a host with *H3* resistance from *S. tuberosum* ssp. *andigena* CPC2802 showed profound impacts on the syncytium during the response to *G. pallida*. Juvenile development was impeded, or predominantly, only males reached the adult stage. However, it has also been reported that continuous multiplication of *G. pallida* on hosts with this resistance for several generations results in increased multiplication [10,21], which involves the maturation of both adult stages. This selection process in which nematodes that can overcome the host’s resistance over several reproductive cycles, can lead to an increased prevalence of *vir* alleles (or a reduction in *Avr* alleles) within the population and their representation at high frequencies in the final lineages. Given the diverse nature of *G. pallida* present in Europe, it is likely that this selection process is based on preferential reproductive success of a virulent subset of the nematodes present in the population. However, it is also possible that novel mutations can occur during selection [23,26,53]. In either of these cases, such changes should be depicted in their genomic background [22,23,24], and here a better insight into selection of *G. pallida* populations was provided with the use of modern genomic tools.

The speed with which *G. pallida* populations overcome host resistances has been reported to be between six and eight generations [25,26], while others report virulence levels stabilising after 10–12 generations [10,26]. The current study showed that the virulence of populations adapted to resistance derived from *S. vernei* or *H3* from *S. tuberosum* ssp. *andigena* CPC2802 was specifically dependent on the genetic background of the potato genotype (Table 1). Both of these resistances are derived from sources where the resistance is the additive effect of more than one quantitative trait *locus* (QTL) [9], with more QTL with statistical significance reported for *S. vernei* than for *H3* [54,55,56]. In the commercial setting, breakdown of resistance to *G. pallida* has already occurred after several crop cycles with some cultivars with resistance derived from *S. vernei* by certain nematode populations [10,27].

Effectors are key factors in determining virulence on specific resistances. In *G. pallida*, effectors are mainly produced in the oesophageal gland cells and are delivered into the host through the stylet [57,58]. A number of large multigene families encoding effectors have been identified in PCNs, including the glutathione synthetases and the secreted SPRY domain proteins (SPRYSECs) [32,48,59,60]. The latter family contains a highly diverse N-terminus attached to a signal peptide and are expressed in the dorsal gland cells during the early stages of parasitism [29,47,61,62]. It has been shown that this hypervariable surface of the domain is a result of polymorphisms emerging during positive selection events [4,63]. Interestingly, it has been found that [4,63] the SPRY protein RBP-1 is an *Avr* gene in *G. pallida* due to its indirect interaction with the NB-LRR protein Gpa2, resulting in activation of defence responses. This avirulence activity is determined by a single amino acid polymorphism found in the SPRY domain. Consistent with this, members of the SPRY domain family were extensively identified here; six were identified in both downstream analyses as in common (Table 2). Here, it was shown that the SPRY domain gene family responds to the *H3* resistance upon selection pressure. This may reflect the presence of different genotypes (or “introductions”) present in the original Newton population that also prevailed throughout generations. This genetic diversity is also consistent with the presence of different mitochondrial DNA types of *G. pallida* within the same field [17].

Other genes for which little information about their function in PCN exists are also identified as being under selection pressure (Table 2). Specifically, Gene Ontology (GO) annotation suggested that orthologues of the gene *SSU72* (similar to the annotated gene *GPALN_009697*) involved in mRNA processing in humans, and the protein unc-80 (similar to *GPALN_013052*) has been suggested to regulate neuronal activity in *Caenorhabditis elegans* upon hormonal reception from its environment [64]. Finally, members of the TWiK family K^+^ channels (similar to *GPALN_004129*) have been found to be differentially expressed in *G. pallida* eggs upon their exposure to root diffusates [65].

The study of the allele frequency of the polymorphisms identified in the re-sequenced samples on either resistance revealed a complete drop of the reference allele in 48 positions of 30 genes in total (Supplementary Table S6). This indicates that the selected alleles, which are mostly a result of SNPs, may be significantly beneficial for the virulence of the specific population and that their absence may affect their recognition by the host (i.e., detection avoidance). However, this can also be a result of either a bottleneck effect during the selection process or allelic dropout during sequencing. Some of these genes are orthologues of known effectors, including SPRYSEC proteins and dorsal gland cell genes of unknown function (Supplementary Table S6). Such sequences represent candidate *Avr* genes along with those identified in both genomic methods (Table 2) and investigation of their functional activity in provoking a response in the host is a priority for future work.

The availability of a greatly improved genome assembly for the *G. pallida* Newton population from which the selected lines were derived, has allowed us to examine the distribution of the polymorphisms in the *G. pallida* genome. This analysis showed that some regions of the genome (Figure 3) are under increased selection pressure and that the regions being targeted were different following selection on the different resistance sources. The presence of certain variable gene families in these regions seems to facilitate nematode populations overcoming resistance barriers, but some could also be a result of linkage drag. Furthermore, specific effector families may respond differently depending on the resistance source as well as the timing of the activated immune response. For instance, cellulases are mostly expressed during the penetration and invasion of the infective J2 through the host cells, whereas SPRYSECs can be expressed during the post-infection and sedentary stages [30,32,48,66].

In this study, unique *G. pallida* populations selected for virulence were used in to measure their reproductive ability on two economically important resistance sources. Our analysis of the histology of the *H3* resistance response adds to our understanding of the mechanisms activated upon nematode infection. Furthermore, using modern genomic techniques combined with sequencing technologies, candidate *Avr* genes were identified for future functional validation assays, while more light was shed on the genomic impact that selection has on nematode populations.

## 5. Conclusions

Continuous multiplication of *G. pallida* on specific resistances creates high selection pressure and can negatively affect the durability of resistance. Understanding the mechanisms underlying selection evolution, as well as effector genes recognised by a specific *R* gene is crucial for the development of diagnostic markers that can inform sustainable deployment of resistance in the field. Modern genomic tools can facilitate this goal and by exploiting the acquired knowledge, the development of new durable resistances in potato will be possible to meet future global demand.

## Figures and Tables

**Table 1:** The mean number of females of *G. pallida* Newton population and its associated selected populations on the root surface of cv. Désirée and Sv_8906, Sv_11305, Sa_11415 and Sa_12674. The experiment was repeated twice with four biological replicates per experiment. The underlined numbers show the mean number of females when the nematode populations were selected on the same resistance source as the tested potato line. The superscripted letters indicate the least significant difference based on Tukey’s test (*p* = 0.05) within each population along the different tested potato lines (i.e., different letters indicate values with statistically significant differences).

	“Désirée”	Sv_8906	Sv_11305	Sa_11415	Sa_12674
Newton	141.0 ± 30.3 ^a^	23.3 ± 0.7 ^b^	23.5 ± 3.8 ^b^	28.4 ± 6.2 ^b^	13.1 ± 3.3 ^b^
n-11305	150.4 ± 18.9 ^a^	74.5 ± 5.0 ^c^	107.0 ± 10.5 ^b^	43.5 ± 2.3 ^c^	4.9 ± 0.3 ^d^
n-11415	84.4 ± 13.2 ^a^	5.1 ± 3.4 ^c^	9.9 ± 2.5 ^c^	109.0 ± 6.2 ^a^	34.6 ± 10.0 ^b^

**Table 2:** Annotated genes identified in common in both PenSeq and ReSeq variant calling analyses. The third column indicates the annotated gene in the current *G. pallida* genome [30] assembly as generated by BLASTp search. In the two genes marked with an asterisk (*), the variant calls were not found in the same populations between the two analyses (possibly as an artefact result and not as candidate *Avr* genes).

Common Genes	Description	Similarity to Gene Annotated in [27]
GPALN_004071	Unknown function	GPLIN_001153200
GPALN_004129	TWiK family of potassium channels	GPLIN_001292400
GPALN_015295	SPRY domain protein	GPLIN_000507800
GPALN_007439	SPRY domain protein	GPLIN_000800200
GPALN_009681	SPRY domain protein	GPLIN_000909700
GPALN_009697	RNA polymerase II subunit A C-terminal domain phosphatase SSU72	GPLIN_001349800
GPALN_012007	SPRY domain protein	GPLIN_000437400
GPALN_012018	SPRY domain protein	GPLIN_000413600
GPALN_013052	Protein unc-80	GPLIN_000425400
GPALN_013436	SPRY domain protein	GPLIN_000433800
GPALN_015286	Unknown function	GPLIN_000807100
GPALN_010968 *	BTB/POZ domain-containing protein 3	GPLIN_000507800
GPALN_006664 *	SPRY domain protein	GPLIN_000716900

## Supplementary Materials

The following are available online at https://www.mdpi.com/2073-4425/11/12/1429/s1, Figure S1: Comparative relative quantification (RQ) of the three known effector genes (SPRY-414-2, SPRY-1719-1 and G16H02) in the pre- and post-enrichment libraries, Figure S2: PenSeq analysis of candidate effector genes on the tested *G. pallida* populations. Table S1: List of effector-encoding *G. pallida* genes and their sequences targeted in PenSeq based on the genome assembly published by Cotton, Lilley [30], Table S2: The primers used for amplifying the three known *G. pallida* effector genes as well as the endogenous control (non-effector) *GAPDH*, Table S3: All-type variant calls (including synonymous changes) identified in PenSeq analysis, Table S4: Variant calls (high and moderate impact) identified in PenSeq analysis after filtering steps. Table S5: All-type variant calls (including synonymous changes) identified in ReSeq analysis. Table S6: Variant calls (high and moderate impact) identified in ReSeq analysis after filtering steps. Supplementary Script: The JAVA-based script used for rendering the distribution of variant calls in both PenSeq and ReSeq. Script has been written by Etienne Lord, Agriculture and Agri-Food Canada, St-Jean-sur-Richelieu, QC Canada) and built with Processing (https://processing.org/).
