# Supplementary material for: The Genomic Impact of Selection for Virulence against Resistance in the Potato Cyst Nematode, Globodera pallida
Source: Genes (Basel). 2020 Nov 28;11(12):1429. doi: 10.3390/genes11121429 (PMC7760817; doi:10.3390/genes11121429)
Supplement: Supplementary file 1 [file genes-11-01429-s001.zip › Supplementary data/genes-985593 supplementary.docx]

Supplementary Table S1. List of effector-encoding *G. pallida* genes and their sequences targeted in PenSeq based on the genome assembly published by [Cotton, Lilley [30]](#_ENREF_30).

Supplementary Table S2. The primers used for amplifying the three known *G. pallida* effector genes as well as the endogenous control (non-effector) *GAPDH.*

| Gene | Forward primer | Reverse primer |
| --- | --- | --- |
| *GAPDH* | GTGATTAGCAACGCTTCGTG | GTCATCAGCCCTTCGATGAT |
| *SPRY-414-2* | GCCAAGGTTACAGGAAAGAA | TTTGTTTGGTCGCAAGTCCA |
| *SPRY-1719-1* | AGAGAAAGGAGAGCACAACG | TTTGAGTATGCGTAAGTGCC |
| *G16H02* | GTCGTTCTCCGTCATTTTGG | GGAAAGCGTGTGAAAGGCAC |

**Supplementary Script. The JAVA-based script used for rendering the distribution of variant calls in both PenSeq and ReSeq. Script has been written by Etienne Lord, Agriculture and Agri-Food Canada, St-Jean-sur-Richelieu, QC Canada) and built with Processing (**[**https://processing.org/**](https://processing.org/)**).**


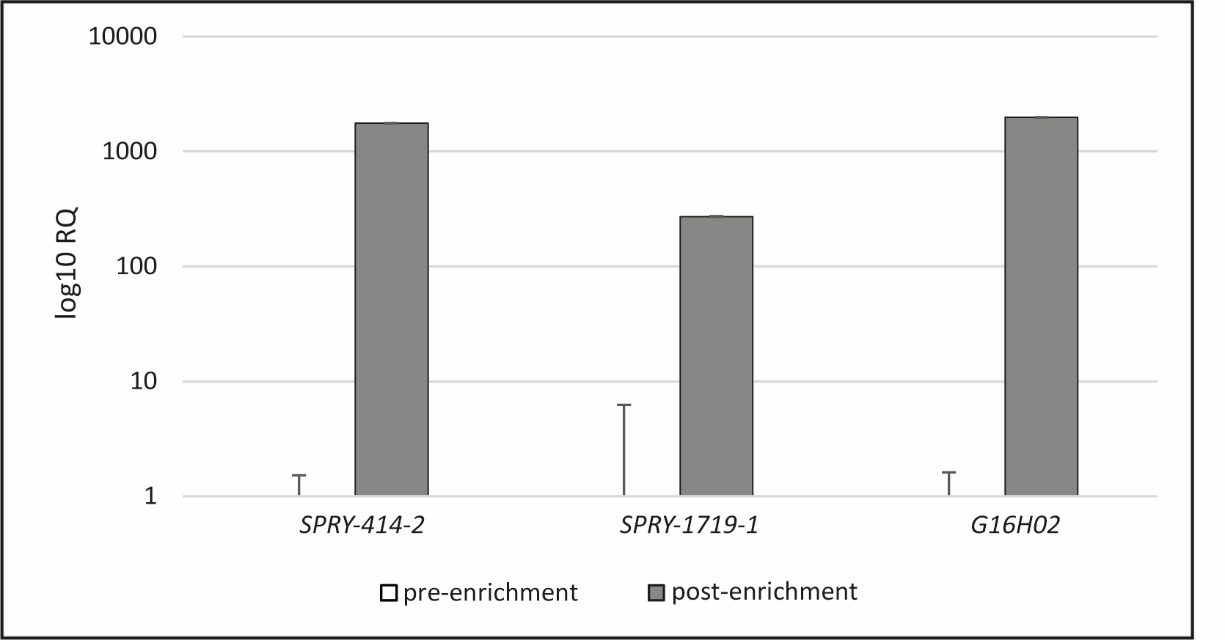


Supplementary Figure S1. Comparative relative quantification (RQ) of the three known effector genes (*SPRY-414-2*, *SPRY-1719-1* and *G16H02*) in the pre- and post-enrichment libraries. The non-effector gene *GAPDH* was used as endogenous control. Error bars stand for 95% confidence interval (CI). RQ values are presented in a logarithmic scale log10.

Supplementary Table S3. All-type variant calls (including synonymous changes) identified in PenSeq analysis. The first two columns show the position of the flagged variant (Scaffold, Position) and the specific nucleotides change in the reference (REF) and alternate (ALT) allele. The SnpEff_INFO column shows the type of the change based on the SnpEff annotation (including the gene transcript with the best BLASTp hit). The last 10 columns indicate the presence of the reference or alternate allele (REF/ALT) in the specific populations.

Supplementary Table S4. Variant calls (high and moderate impact) identified in PenSeq analysis after filtering steps. The first two columns show the position of the flagged variant (“Scaffold”, “Position”) and the specific nucleotides change between the reference (REF) and alternate (ALT) allele. In the columns “Gene”, “Description”, “Length” and “e.Value”, the gene with the best BLASTp hit is shown along with the BLAST e-value result and the InterPro Gene Ontology flagged by Blast2Go (“InterPro.GO.Names”). The presence of the reference or alternate allele (REF/ALT) in the specific populations are presented, combined with the resistance source where the ALT allele was identified in (“Selection”).

Supplementary Table S5. All-type variant calls (including synonymous changes) identified in ReSeq analysis. The first two columns show the position of the flagged variant (Scaffold, Position), the type and the specific nucleotides change in the reference (REF) and alternate (ALT) allele. The SnpEff_INFO column shows the type of the change based on the SnpEff annotation (including the gene transcript with the best BLASTp hit). The presence of the REF/ALT allele in each population is presented along with their AF values.

Supplementary Table S6. Variant calls (high and moderate impact) identified in ReSeq analysis after filtering steps. The first two columns show the position of the flagged variant (“Scaffold”, “Position”) and the specific nucleotides change between the reference (REF) and alternate (ALT) allele. In the columns “Gene”, “Description”, “Length” and “e.Value”, the gene with the best BLASTp hit is shown along with the BLAST e-value result and the InterPro Gene Ontology flagged by Blast2Go (“InterPro.GO.Names”). The presence of the reference or alternate allele (REF/ALT) in the specific populations are presented, combined with the resistance source where the ALT allele was identified in (“Selection”). Variant calls highlighted with pale red are associated with both resistance sources. In the column “Other”, the variant called homozygote (i.e., AF = 0) for either resistance source is shown.

Supplementary Figure S2. PenSeq analysis of candidate effector genes on the tested *G. pallida* populations. Each box represents an entire RBBH coding sequence of a gene (including their exons) (*x*-axis). The *y*-axis indicates the coverage of the targeted gene on a log-scale. Each line shows the coverage of a certain nematode population.
