# Supplementary material for: The Genomic Impact of Selection for Virulence against Resistance in the Potato Cyst Nematode, Globodera pallida
Source: Genes (Basel). 2020 Nov 28;11(12):1429. doi: 10.3390/genes11121429 (PMC7760817; doi:10.3390/genes11121429)
Supplement: Supplementary file 1 [file genes-11-01429-s001.zip › Supplementary data/supplementary table 2.docx]

Supplementary Table 2. The primers used for amplifying the three known *G. pallida* effector genes as well as the endogenous control (non-effector) *GAPDH.*

| Gene | Forward primer | Reverse primer |
| --- | --- | --- |
| *GAPDH* | GTGATTAGCAACGCTTCGTG | GTCATCAGCCCTTCGATGAT |
| *SPRY-414-2* | GCCAAGGTTACAGGAAAGAA | TTTGTTTGGTCGCAAGTCCA |
| *SPRY-1719-1* | AGAGAAAGGAGAGCACAACG | TTTGAGTATGCGTAAGTGCC |
| *G16H02* | GTCGTTCTCCGTCATTTTGG | GGAAAGCGTGTGAAAGGCAC |
