# Supplementary figures and images for: The Genomic Impact of Selection for Virulence against Resistance in the Potato Cyst Nematode, Globodera pallida
Source: Genes (Basel). 2020 Nov 28;11(12):1429. doi: 10.3390/genes11121429 (PMC7760817; doi:10.3390/genes11121429)

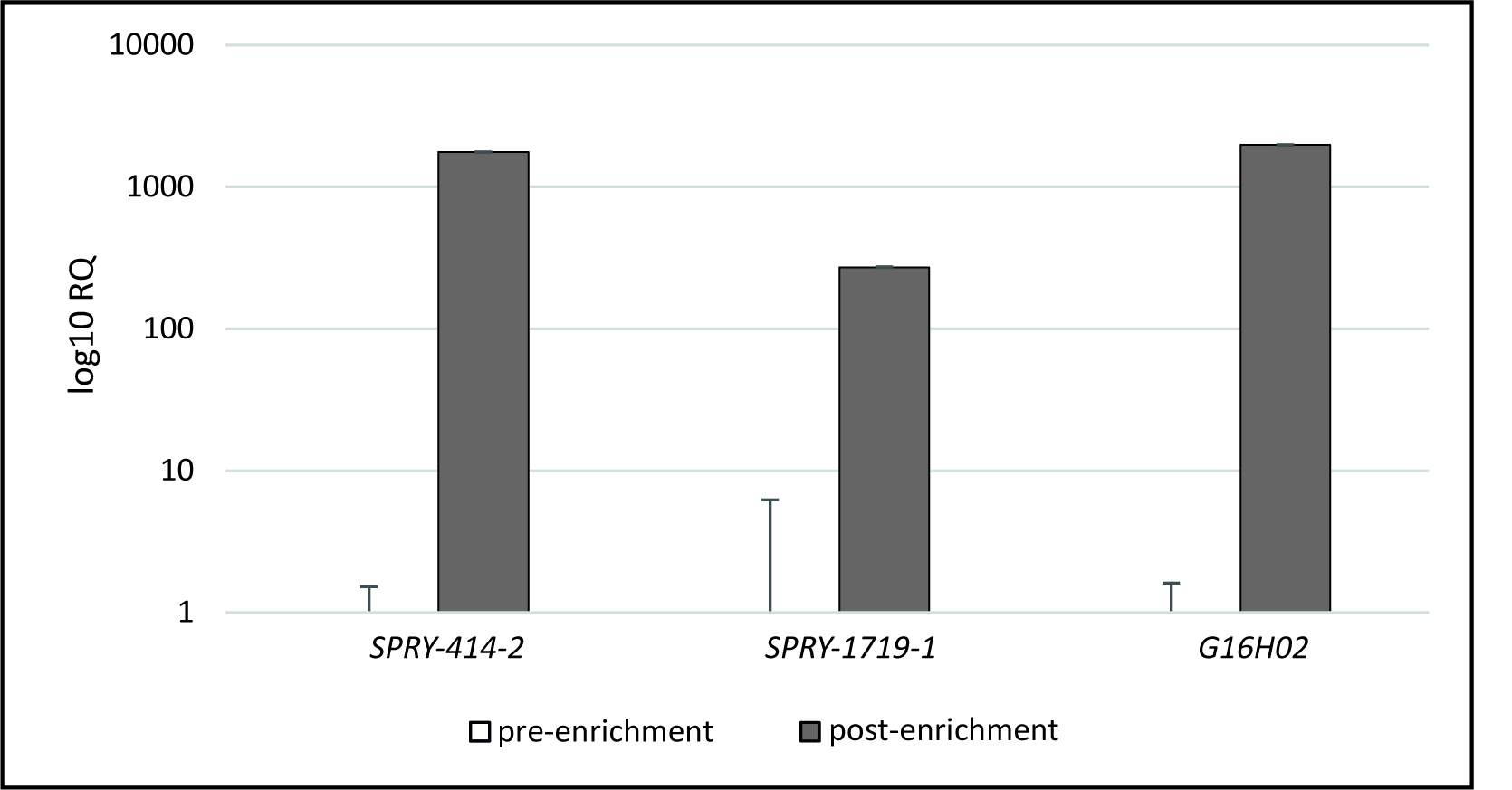

Supplement: Supplementary file 1 [file genes-11-01429-s001.zip › Supplementary data/Supplementary Figure 1-qpcr_results.tif]

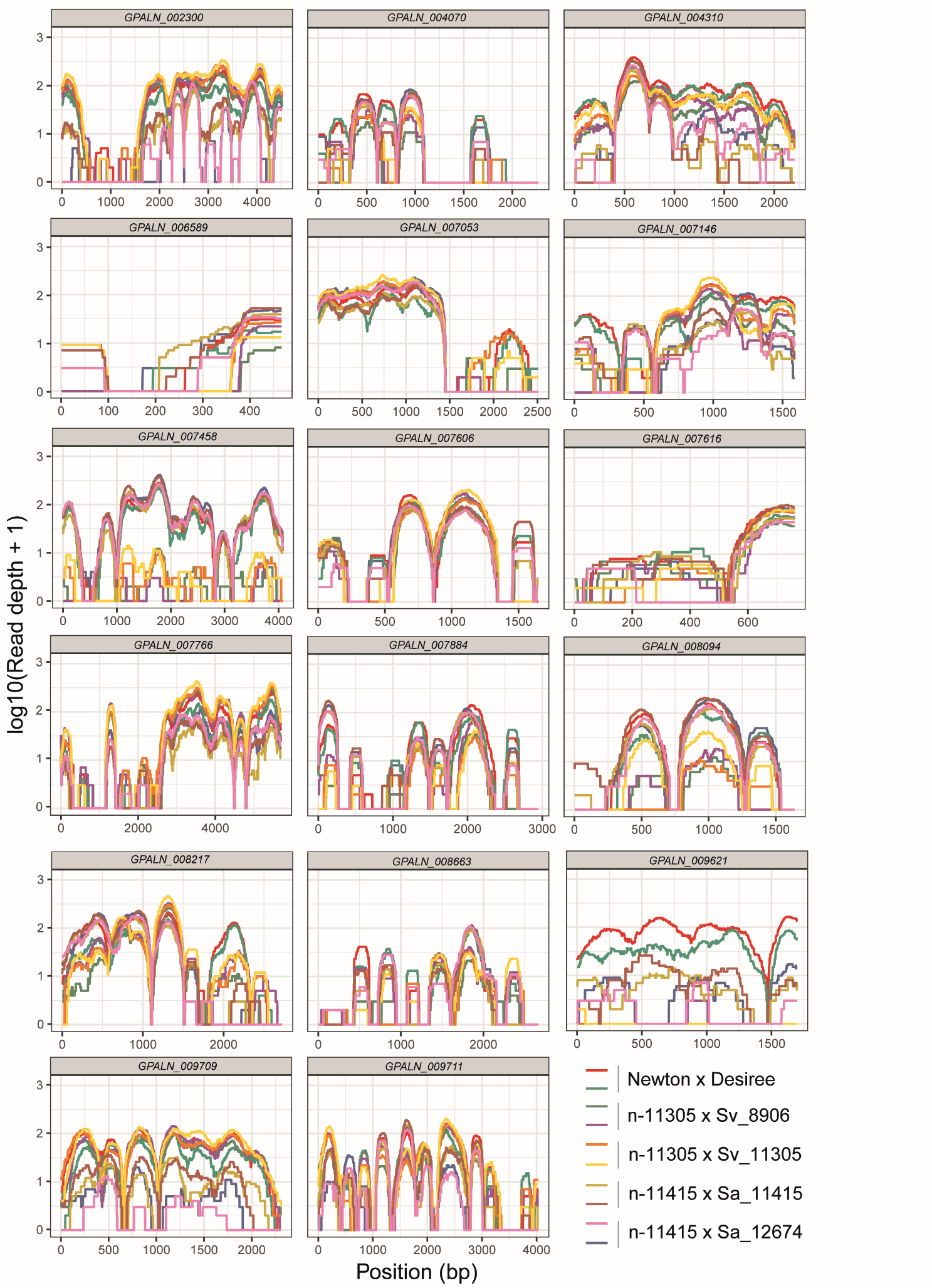


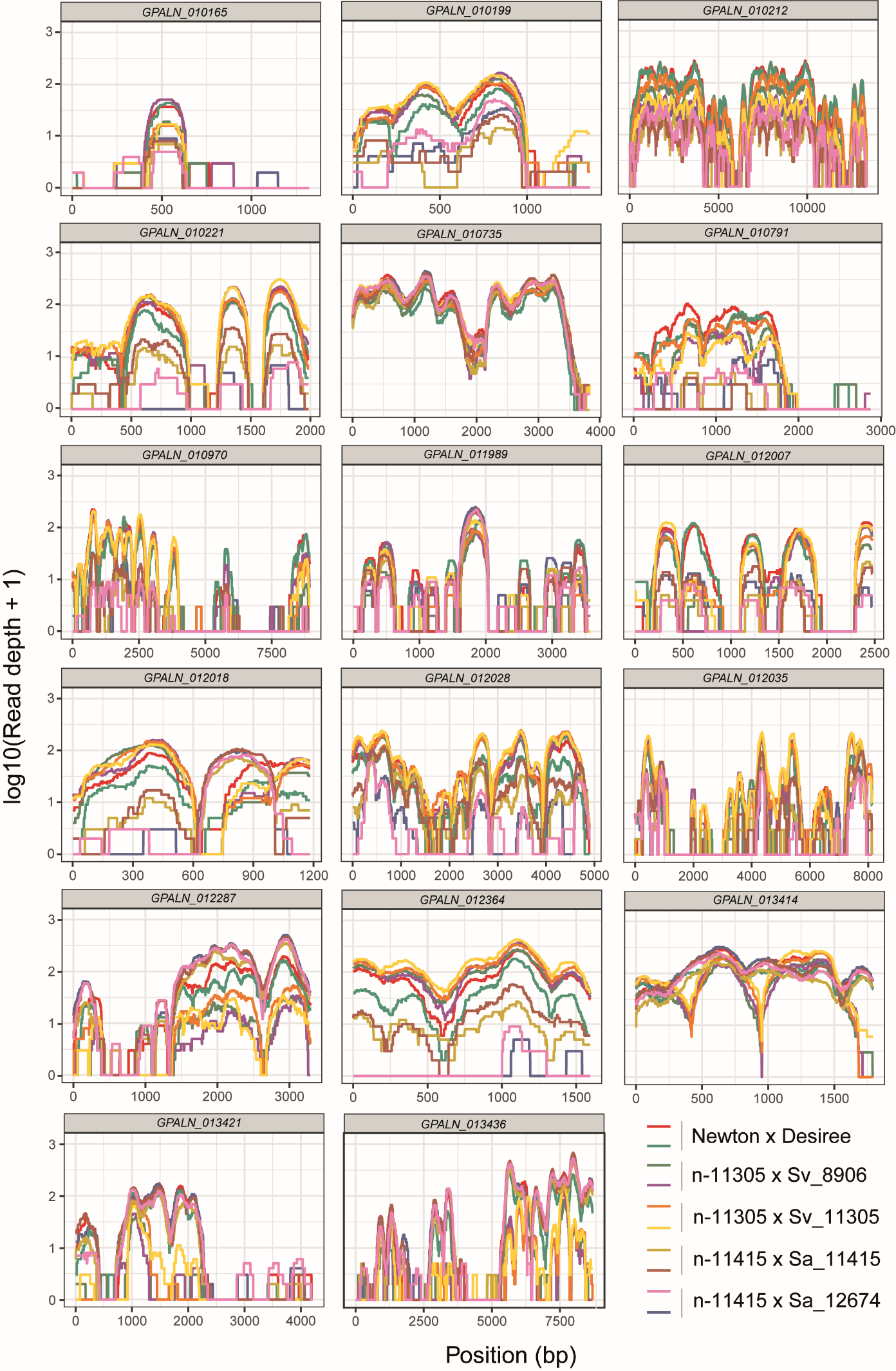


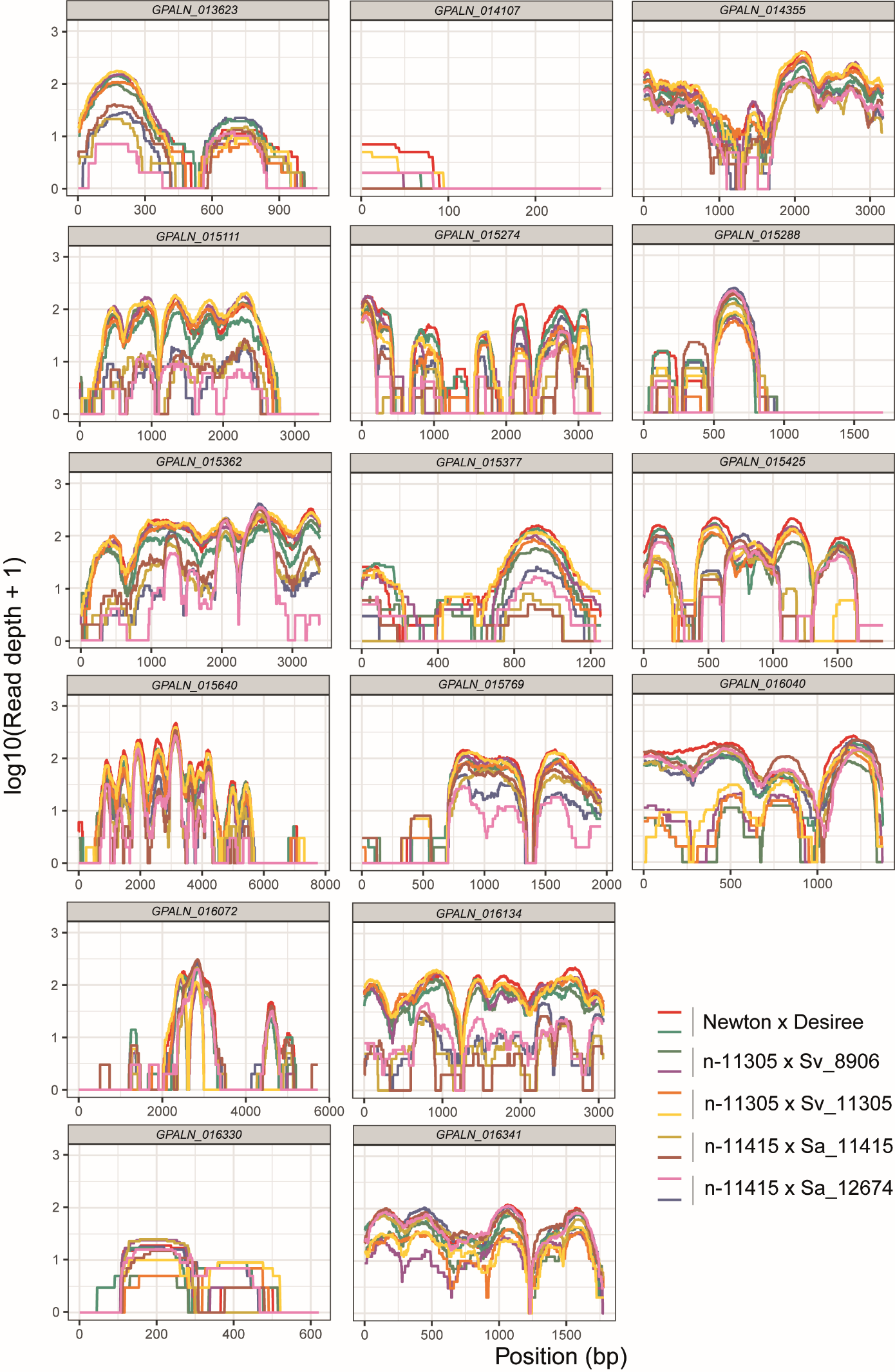

Supplement: Supplementary file 1 [file genes-11-01429-s001.zip › Supplementary data/Supplementary Figure 2-penseq_coverage.docx]
